# Small Subset, Big Impact: Regulatory Function of γδ T Cells in Arteriogenesis

**DOI:** 10.3390/cells15080709

**Published:** 2026-04-17

**Authors:** Kira-Sofie Wimmer, Carolin Baur, Matthias Kübler, Christoph Arnholdt, Konda Kumaraswami, Franziska Heim, Katharina Elbs, Michael Reha Rohrmoser, Daphne Merkus, Elisabeth Deindl

**Affiliations:** 1 Institute of Surgical Research at the Walter-Brendel-Centre of Experimental Medicine, University Hospital, Ludwig-Maximilians-Universität München, 81377 Munich, Germany; kira.wimmer@med.uni-muenchen.de (K.-S.W.); carolin.baur@med.uni-muenchen.de (C.B.); matthias.kuebler@med.uni-muenchen.de (M.K.); christophjohannes.arnholdt@med.uni-heidelberg.de (C.A.); franziska.heim@med.uni-muenchen.de (F.H.); katharina.elbs@med.uni-muenchen.de (K.E.); michael.rohrmoser@med.uni-muenchen.de (M.R.R.); daphne.merkus@med.uni-muenchen.de (D.M.); 2 Biomedical Center, Institute of Cardiovascular Physiology and Pathophysiology, Faculty of Medicine, Ludwig-Maximilians-Universität München, 82152 Planegg-Martinsried, Germany; 3 Deutsches Zentrum Immuntherapie (DZI) and Comprehensive Cancer Center Erlangen-EMN (CCC ER-EMN), Friedrich-Alexander-Universität Erlangen-Nürnberg (FAU), 91054 Erlangen, Germany; 4 Department of Oral- and Cranio-Maxillofacial Surgery, Friedrich-Alexander-Universität Erlangen-Nürnberg (FAU), 91054 Erlangen, Germany; 5 Department of Ophthalmology, University of Heidelberg, 69120 Heidelberg, Germany; 6 Immunoregulation Section, Laboratory of Molecular Biology and Immunology, National Institute on Aging, 251 Bayview Blvd, Suite 100, Baltimore, MD 21224, USA; 7 Division of Experimental Cardiology, Department of Cardiology, Thoraxcenter, Erasmus MC, University Medical Center Rotterdam, 3015 Rotterdam, The Netherlands

**Keywords:** αβ T cells, cardiovascular, collateral artery growth, IFNγ, IL-10, macrophages, mast cells, neutrophils, peripheral artery disease, lymphocytes, γδ T cells

## Abstract

**Highlights:**

**What are the main findings?**

- γδ T cells are indispensable for perfusion recovery and collateral vessel growth, whereas αβ T cells are not
- Different γδ T cell subtypes sequentially modulate arteriogenesis via IFNγ and IL-10

**What are the implications of the main findings?**

- γδ T cells are capable of orchestrating crosstalk between the immune and vascular systems
- The capacity of γδ T cells to modulate immune mediators may extend their regulatory role to other inflammatory processes

**Abstract:**

Despite the identification of several mediators of arteriogenesis, the growth of natural bypass, the role of lymphocytes, particularly T cells, in this process remains poorly defined. Among these, γδ T cells, which express alternative T cell receptors, have emerged as a key immune component. This study examined the roles of αβ and γδ T cells in arteriogenesis using a murine hindlimb model. While the absence of αβ T cells did not affect arteriogenesis, γδ T cell depletion markedly reduced vascular cell proliferation and perfusion recovery. Early phase analyses revealed impaired mast cell activation, whereas platelet–neutrophil aggregates and neutrophil extravasation were unaffected. In the later proliferative phase, γδ T cell depletion hindered perivascular M2-like (MRC1^+^) macrophage accumulation. Flow cytometric analysis of whole blood in wildtype mice revealed a temporal shift in γδ T cell populations from a CD27^+^/CD39^−^ phenotype, commonly associated with pro-inflammatory functions and IFNγ production, to CD39^+^ phenotypes, which have been linked to anti-inflammatory properties and IL-10 production. In rescue experiments, administration of IFNγ to γδ T cell-depleted mice restored mast cell activation, whereas IL-10 treatment reestablished M2-like (MRC1^+^) macrophage accumulation. These findings collectively identify γδ T cells as critical regulators of both early and late phases of arteriogenesis through coordinated inflammatory and regenerative mechanisms.

## 1. Introduction

Cardiovascular diseases (CVDs), primarily driven by arterial occlusions that impair tissue perfusion, remain the main causes of morbidity and mortality worldwide [1]. While revascularization procedures such as bypass surgery and percutaneous interventions are effective, they are not universally feasible and carry substantial procedural risks, particularly in multimorbid patient populations.

Arteriogenesis—the growth of pre-existing arterial collaterals through remodeling and vascular cell proliferation in response to shear stress—represents a promising conservative therapeutic approach, which mainly relies on actions of the immune system [2]. Despite its therapeutic potential, no clinically approved pharmacological approaches to enhance arteriogenesis are currently available.

To develop drugs efficaciously promoting collateral bypass growth, it is of the utmost importance to examine the driving mechanisms on a molecular level. Arteriogenesis is induced by increased shear stress in pre-existing arterioles following arterial occlusion of a main artery. This triggers endothelial activation, release of von Willebrand factor (VWF), and upregulation of adhesion molecules and chemokines [3,4,5]. VWF-activated platelets interact with neutrophils, causing them to produce reactive oxygen species (ROS), which is necessary for mast cell activation [6,7,8,9]. Through the release of histamine and other mediators, mast cells enhance vascular permeability and recruit monocytes to the perivascular space. Those monocytes then differentiate into macrophages, which undergo early polarization towards inflammatory M1-like macrophages and subsequently to regenerative M2-like macrophages, driving vascular remodeling and cell proliferation [10,11,12,13]. Proliferating vascular cells then act as direct effectors of lumen expansion and structural remodeling characteristic of functional collateral growth [6,14].

The involvement of multiple cellular mediators in arteriogenesis is well established. However, given that arteriogenesis constitutes a process driven by the immune system, the contribution of immune cell subsets, such as T cells, remains insufficiently characterized. Although T cells are central components of adaptive immunity and frequently exert regulatory functions, emerging evidence suggests that they may also participate in trained innate immune responses [15,16]. Among these, γδ T cells constitute a unique subset that bridges innate and adaptive immunity, characterized by rapid activation and potent cytokine secretion [17]. Through their capacity to modulate local inflammatory and angiogenic processes, γδ T cells are well positioned to potentially influence vascular remodeling in a broader context [18,19,20].

Previous research on γδ T cells has largely centered on their dual roles in the tumor microenvironment, where both pro- and anti-tumorigenic functions have been described [21,22]. In various autoimmune disease models, γδ T cells have been reported to exert either pathogenic or protective functions, largely determined by their cytokine profiles [23,24,25,26]. In models of arterial plaque formation, γδ T cells have been shown to enhance plaque stability while promoting inflammation through cytokine release and induction of cell death [27]. However, despite the functional parallels of these processes, their role in arteriogenesis and collateral vessel growth has not yet been investigated.

In this study, we therefore employed a murine hindlimb model of arteriogenesis to evaluate the impact of αβ T cells and γδ T cells on perfusion recovery, vascular proliferation, and perivascular immune cell activation, respectively comparing TCRα knockout mice or γδ T cell-depleted mice with control mice.

## 2. Materials and Methods

All procedures were approved by the Bavarian Animal Care and Use Committee (Regierung Oberbayern; ethical approval code: ROB-55.2Vet-2532.Vet_02-17-99; approved on 8 December 2017 and Vet_02-22-99; approved on 29 March 2023) and conducted in accordance with the corresponding animal protocol and German animal welfare regulations.

### 2.1. Animals

Male C57BL/6 mice (8–12 weeks of age; Charles River Laboratories, Wilmington, MA, USA) and male T cell receptor α knockout mice (TCRα KO, 8-12 weeks of age; B6.129S2-Tcratm1Mom/J, strain #002116, Jackson Laboratory, Bar Harbor, ME, USA) were housed under standard laboratory conditions with ad libitum access to enrichment, food, and water. Animals whose health status was compromised were excluded based on predefined criteria assessed through daily monitoring using standardized checklists. According to this assessment, one mouse had to be excluded before surgical intervention. Mice were allocated to experimental groups in a random manner. To minimize baseline variability, animals were matched as closely as possible for age, sex, and body weight prior to group assignment. For laser Doppler imaging, flow cytometry, and staining, *n* = 5 mice per group were used, aligned with prior power calculations. In selected control conditions, group sizes of *n* = 3–4 were considered sufficient based on prior reproducibility and reduction principles. All experimental procedures were performed in accordance with ARRIVE guidelines, institutional, and national guidelines.

### 2.2. γδ T Cell Depletion and Control Treatments

To deplete γδ T cells, mice received an anti-mouse TCR γ/δ antibody (clone UC7-13D5, 107517, BioLegend, San Diego, CA, USA) at a dosage of 9 mg/kg body weight by intravenous injection via the tail vein 24 h before surgery. The selected dose was based on manufacturer recommendations and published application data demonstrating effective depletion over the required timeframe [28,29]. Sustained depletion was validated by flow cytometry analysis at the experimental endpoint (day 7). To control for potential immunological effects of the antibody, a separate group received the corresponding IgG isotype control antibody (clone 400940, BioLegend, San Diego, CA, USA), also at a dosage of 9 mg/kg under the same conditions, 24 h before femoral artery ligation.

### 2.3. Surgical Induction of Arteriogenesis

Arteriogenesis was induced by ligating the femoral artery distal to the origin of the circumflex branch with a surgical suture after separating it from the adjacent nerve and vein (Supplementary Materials Figure S1) [30]. On the contralateral side, a sham operation without ligation served as an internal control.

For anesthesia, mice received a subcutaneous injection of MMF (fentanyl 0.05 mg/kg, CuraMED Pharma (Paterson, NJ, USA); midazolam 5 mg/kg, Ratiopharm GmbH (Ulm, Germany); medetomidine 0.5 mg/kg, Pfizer Pharma (New York City, NY, USA)). Depth of anesthesia was verified by loss of the interdigital reflex. Mice were maintained on a 37 °C warming pad throughout surgery. Buprenorphine (0.1 mg/kg, Dechra Veterinary Products (Northwich, UK)) was administered subcutaneously 10 min prior to antagonization of the anaesthesia for analgesia and every 8 h until day 3 post-surgery.

On day 3 after FAL, the animals were anaesthetized by continuous administration of 1.5% isoflurane; at all other times, the animals were anaesthetized by subcutaneous injection of MMF as described before.

### 2.4. In Vivo Treatments

#### 2.4.1. IFNγ Treatment

Recombinant interferon γ (IFNγ) substitution was performed by intravenous injection of 1 µg IFNγ (575308, BioLegend; equivalent to 40 µg/kg body weight, diluted in 100 µL sterile saline) via the tail vein. Injections were initiated immediately after femoral artery ligation (FAL) and repeated every 12 h during the first 3 days, followed by once-daily injections on days 4 and 5.

#### 2.4.2. IL-10 Treatment

Interleukin-10 (IL-10) treatment was conducted by administering a single intravenous dose of 0.5 µg IL-10 (575802, BioLegend; 20 µg/kg body weight, diluted in 100 µL sterile saline) via the tail vein on day 3 after femoral artery ligation. The dosage was adopted from a previously published study [31], whereas the time point of administration was determined based on our own experimental data to coincide with the observed shift in γδ T cell subtype expression during collateral vessel growth.

### 2.5. Perfusion Assessment by Laser-Doppler Imaging

Laser-Doppler imaging (Moor LDI 5061, Moor Instruments, Axminster, UK; software version 3.01) was used to assess perfusion recovery. Baseline measurements were taken before surgery, immediately after ligation to confirm occlusion of the femoral artery, and on days 3 and 7 post-surgery. 

To exclude external influences such as warmer ambient temperature and possible vasoconstricting effects of the used anesthetics, the measurement was carried out over an acclimatization period of 10 min in each case. To evaluate the measurement results, an area of exactly 0.42 cm^2^ was defined on each paw, and the perfusion of the sham side was set as the reference value, to which the percentages of the operated side referred (Supplementary Materials Figure S2).

### 2.6. Tissue and Blood Collection

On days 1, 3, or 7 post-surgery, mice were anesthetized with MMF and euthanized by cervical dislocation. To stabilize the vascular walls for tissue processing, the aorta was cannulated, and hindlimbs were perfused sequentially with 1% adenosine in phosphate-buffered saline (PBS, Sigma-Aldrich, St. Louis, MO, USA), containing 5% bovine serum albumin (BSA, Sigma-Aldrich), followed by 3% paraformaldehyde (PFA, Merck, Darmstadt, Germany). On day 1, samples were fixed directly in 3% PFA for 1 h without prior perfusion. 

Adductor muscles were harvested bilaterally, cryoprotected in 15% sucrose (1 h) and 30% sucrose (overnight), embedded in Tissue-Tek compound (Sakura Finetek, Torrance, CA, USA), and stored at −80 °C. Whole blood was collected via cardiac puncture and heparinized for flow cytometry analysis.

### 2.7. Immunofluorescence Staining

Cryosections (8 µm) were prepared, focusing on collaterals in the adductor muscle (Supplementary Materials Figure S3). Imaging was performed with a Leica DM6 B epifluorescence microscope (Leica Microsystems, Wetzlar, Germany). For all tissue analyses using immunofluorescence staining, three tissue sections with two collaterals each were evaluated per muscle sample. Depending on the cell population or parameter assessed, either the collateral vessel itself or the immediate surrounding perivascular space was analyzed.

#### 2.7.1. Macrophage Staining

Sections were fixed with 4% PFA, blocked with 10% donkey serum, and incubated overnight at 4 °C with anti-MRC1 (ab64693, Abcam, Cambridge, UK; 1:200). Secondary detection was performed with Donkey anti-Rabbit Alexa Fluor 546 (A-10040, Thermo Fisher, Waltham, MA, USA; 1:200). After blocking with 4% BSA, sections were incubated with anti-CD68 Alexa Fluor 488 (ab201844, Abcam; 1:200), anti-CD31 Alexa Fluor 647 (102516, BioLegend; 1:100), and DAPI (62248, Thermo Fisher; 1:1000). Macrophage subsets were identified based on CD68 and MRC1 expression as commonly used markers for macrophage presence and M2-like phenotypes [8,32,33]. While this approach does not allow a comprehensive phenotypic characterization, it enables an approximate assessment of macrophage infiltration and the direction of polarization (pro-inflammatory M1-like or regenerative M2-like phenotypes) during arteriogenesis in γδ T cell-depleted mice.

#### 2.7.2. 5-Bromo-2′-Deoxyuridine (BrdU) Staining

Mice received daily intraperitoneal injections of BrdU (1.25 mg in 100 µL PBS/0.1% Tween-20/0.5% BSA) beginning directly after FAL. Sections were denatured with HCl (30 min, 37 °C), permeabilized with 0.2% Triton X-100, and blocked with 10% goat serum (Abcam). Anti-BrdU (ab6326, Abcam; 1:50) was applied overnight at 4 °C, followed by Goat anti-Rat Alexa Fluor 546 (A11081, Thermo Fisher; 1:100). Sections were counterstained with CD31 Alexa Fluor 647 (102516, BioLegend; 1:100), ACTA2 Alexa Fluor 488 (F3777, Sigma-Aldrich; 1:400), and DAPI.

#### 2.7.3. Myeloperoxidase (MPO) Staining

Sections were fixed in 4% PFA, permeabilized with 0.2% Triton X-100, and blocked with 10% donkey serum (Abcam), and incubated overnight with anti-myeloperoxidase (MPO, AF3667, R&D Systems, Minneapolis, MN, USA; 1:100). Secondary detection was performed with Donkey anti-Goat Alexa Fluor 594 (A11058, Invitrogen, Waltham, MA, USA; 1:100). Sections were then incubated with anti-mouse ACTA2 Alexa Fluor 488 (F3777, Sigma-Aldrich; 1:400) and DAPI.

### 2.8. Giemsa Staining

Sections were fixed in 4% PFA for 5 min, stained with Giemsa solution (Carl Roth GmbH, Karlsruhe, Germany; 1:25 in distilled water, 1 h at 65 °C), rinsed in 0.5% acetic acid, dehydrated in graded ethanol (96%, 99%), and cleared in xylene. Images were acquired with a Leica DM6 B microscope (Leica Microsystems, Wetzlar, Germany) at 40× magnification. For tissue analysis using this staining technique, 5 sections with 2 collaterals each were evaluated per muscle sample.

### 2.9. Flow Cytometry

Whole blood was collected on day 1, day 3, and day 7 after femoral artery ligation by cardiac puncture into heparinized syringes. Red blood cells were lysed using BD FACS™ Lysing Solution (BD Biosciences, Franklin Lakes, NJ, USA), then leukocytes and platelets were pelleted by centrifugation for 5 min at room temperature with 400 × gravity. All samples were analyzed on a BD LSRFortessa™ Cell Analyzer (BD Biosciences), and compensation controls were included for each fluorochrome. Data was analyzed using the FlowJo v10 software.

#### 2.9.1. Platelet Aggregate Analysis

Cells were stained with anti-CD11b (PE, 1:300, BioLegend), anti-CD115 (BV421, 1:300, BioLegend), anti-CD41 (FITC, 1:400, BioLegend), anti-Gr-1 (APC, 1:800, BioLegend), and Viability Dye (APC-Cy7, 1:1000, Invitrogen) in 1% BSA/PBS. Samples were incubated for 20 min at 4 °C in the dark, washed, and resuspended in 300 µL 1% BSA for acquisition.

#### 2.9.2. γδ T Cell Depletion Analysis

For quantitative confirmation of successful γδ T cell depletion, cells were incubated in the dark at 4 °C for 30 min, dissolved in 200 µL of Viability Dye (eFluor 450, 1:1000, Thermo Fisher) on a 96-well V-bottom plate. The cell suspension was resuspended in 50 µL of Fc-Block, centrifuged again, then stained with anti-CD45 (APC-Cy7, BioLegend) and anti-TCRγ/δ (Brilliant Violet 711, BD Biosciences) at 4 °C in the dark. Finally, the suspension was washed and resuspended in 300 µL of 1% BSA pending acquisition.

#### 2.9.3. γδ T Cell Subset Analysis

For characterization of γδ T cell subsets, cells were stained with anti-CD3 (eFluor450, 1:300, Invitrogen), anti-TCRγ/δ (APC, 1:300, BioLegend), anti-CD39 (PE, 1:100, BioLegend), anti-CD27 (FITC, 1:200, BioLegend), and Viability Dye (APC-Cy7, 1:1000, Invitrogen) in 4% BSA/PBS. Staining was performed at 4 °C in the dark for 20 min. After washing, samples were resuspended in 300 µL 1% BSA and kept on ice until acquisition.

### 2.10. Genotyping of TCRα KO

αβ T cell deficiency was confirmed by genotyping. Genomic DNA was harvested from tissue samples (ear biopsies) using the KAPA HotStart Mouse Genotyping Kit (KK7351, Kapa Biosystems, Wilmington, MA, USA) according to the manufacturer’s instructions. Genotyping was performed using two separate Polymerase Chain Reactions (PCR) to distinguish TCRα (T cell receptor alpha) wildtype and knockout alleles. PCR set A contained Primer 1 (JL-TCR alpha common 5′-TGACTCCCAAATCAATGTGC-3′) and Primer 3 (JL-TCR alpha-knockout-rev, 5′-CCTACCCGCTTCCATTGCTCA-3′), producing a 400 bp amplicon corresponding to the knockout allele. PCR set B contained Primer 1 and Primer 2 (JL-TCR alpha-wildtype-rev, 5′-GGTGAGATGACCCAAAGCAG-3′), producing a 250 bp amplicon corresponding to the wildtype allele. Each PCR (25 µL) contained 10.5 µL nuclease-free water, 12.5 µL KAPA2G Fast HotStart Genotyping Mix (KK5621, Kapa Biosystems), 1.25 µL of each primer, and 1 µL sample DNA. PCR cycling conditions were 95 °C for 3 min; then a total of 35 cycles of 95 °C for 15 s, 60 °C for 15 s, 72 °C for 30 s; and a final extension at 72 °C for 2 min. PCR amplicons were separated on a 1.5% agarose gel with a 100 bp Marker ladder. Genotypes were assigned as follows: a band in PCR set A only indicated TCRα KO, a band in PCR set B only indicated wildtype, and a band in both sets indicated heterozygote allele expression.

### 2.11. Quantitative Real-Time PCR of Ifng (Encoding IFNγ) mRNA Expression in γδ T Cells

For quantitative real-time PCR (RT-qPCR), RNA from sorted γδ T cells was isolated using an RNA extraction kit (RNeasy, 74104, Qiagen, Hilden, Germany) according to the manufacturer’s instructions. RNA was eluted in 30 µL RNase-free water, and purity and concentration were assessed by spectrophotometry (NanoDrop, Wilmington, DE, USA). Complementary DNA (cDNA) was synthesized using the Maxima H Minus First Strand cDNA Synthesis Kit (K1652, Thermo Fisher Scientific) and used as the template for PCR. Quantitative PCR was performed using SYBR Green Master Mix with gene-specific primers for Ifng (forward 5′-GAAAATCCTGCAGAGCCAGA-3′; reverse 5′-CATGAATGCATCCTTTTTCG-3′; amplicon length: 183 bp) and 18S rRNA as a housekeeping gene (forward 5′-GGACAGGATTGACAGATTGATAG-3′; reverse 5′-CTCGTTCGTTATCGGAATTAAC-3′; amplicon length: 108 bp). Reactions were run on a StepOnePlus Real-Time PCR System (Applied Biosystems, Foster City, CA, USA) with annealing temperatures of 60 °C (Ifng) and 64 °C (18S rRNA) for 45 and 40 cycles, respectively. Relative Ifng mRNA expression was determined using the ΔΔCt method after normalization to 18S rRNA.

### 2.12. Differential Blood Analysis

To control for effects of γδ T cell depletion on other leucocyte populations during experiments employing femoral artery ligation, differential blood counts were performed using an automated hematology analyzer (ProCyte Dx, Idexx Laboratories, Westbrook, ME, USA) with mouse-specific settings. Blood was collected via cardiac puncture from anesthetized mice at 24 h after FAL, immediately prior to euthanasia by cervical dislocation.

### 2.13. Statistical Analysis

Sample size was determined a priori using G*Power software (version 3.1.9.2) based on expected effect sizes derived from previous studies employing the same animal model [8,33]. This approach was intended to support adequate statistical power and limit the risk of false-negative results. The primary outcome was perfusion recovery post-surgical intervention, assessed by laser-Doppler imaging. All analyses were performed by a single trained observer manually using identical imaging settings and predefined criteria (consistent ROI size, high marker expression threshold, standardized gating) applied across all samples to reduce the likelihood of systematic bias. Blinding of the primary analyst was not feasible due to the nature of the experimental design; however, all data were independently reviewed by a second investigator who was blinded to the experimental conditions. While inter-observer variability was not formally assessed, these measures were intended to promote consistency and limit potential bias in the analysis. Data are presented as mean ± standard error of the mean (SEM). Group comparisons were performed using one-way or two-way ANOVA followed by Bonferroni’s multiple comparison test, as appropriate. For comparisons between two groups, unpaired Student’s *t*-tests were applied. Data distribution was assessed prior to parametric testing, and no additional effect size measures were calculated. A *p*-value < 0.05 was considered statistically significant. Each datapoint represents the collateral average of the muscle sample. The dotted lines represent the average in sham muscles. Statistical analyses were conducted using GraphPad Prism (version 10, GraphPad Software, San Diego, CA, USA).

## 3. Results

To define the role of T lymphocytes in arteriogenesis, we used a murine hindlimb model of collateral artery growth, in which arteriogenesis was induced by femoral artery ligation [30]. Perfusion recovery, collateral artery remodeling in the adductor muscle, and perivascular leukocyte accumulation and responses were quantified in TCRα KO mice (deficient of αβ T cells) as well as in γδ T cell-depleted, iso-antibody-treated (isotype), and untreated wildtype C57Bl/6 mice.

### 3.1. Deficiency of αβ T Cells Does Not Affect Perfusion Recovery or Vascular Cell Proliferation

To assess the contribution of T cells to collateral vessel growth, we induced arteriogenesis in TCRα KO and corresponding wildtype control mice. Importantly, as an initial control experiment, we found that the absence of conventional αβ T cells, including CD8^+^ cytotoxic and CD4^+^ helper subsets, does not impair perfusion recovery compared with wildtype mice (Supplementary Figure S4a and Ref. [34]). Likewise, vascular cell proliferation and collateral lumen diameter remain unchanged (Supplementary Figure S4b,c and Ref. [34]), indicating that αβ T cells do not contribute to this process.

### 3.2. γδ T Cell Depletion Reduces Hindlimb Perfusion Recovery

To depict the role of γδ T cells in the regulation of arteriogenesis, C57BL/6 mice received a γδ T cell-depleting antibody, while iso-antibody-treated and untreated wildtype mice served as controls. To validate the effect of γδ T cell depletion, additional flow cytometry analyses were performed on depleted versus isotype antibody-treated mice. As a control for potential effects on other leukocyte populations, total white blood cells and their major subsets were quantified using routine hematological analysis.

**Figure 1:**
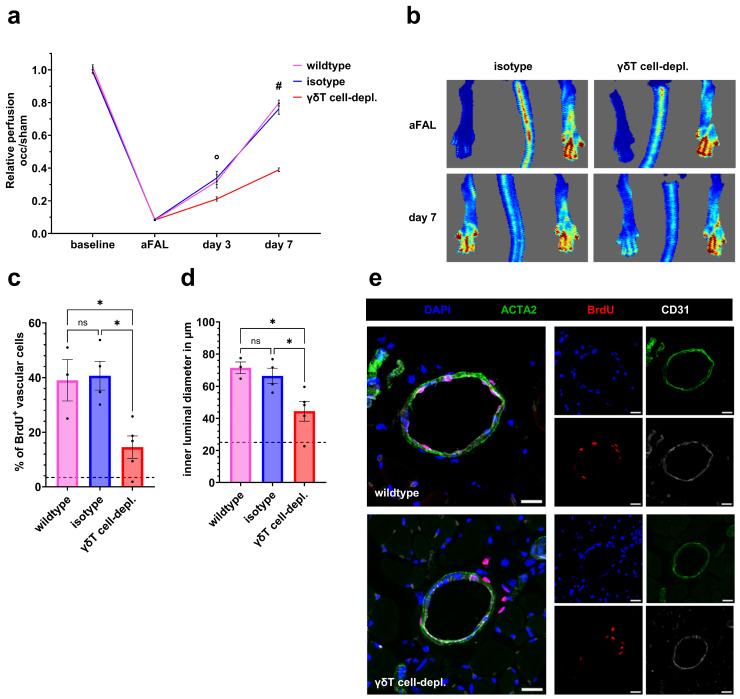
γδ T cell depletion significantly reduces perfusion recovery, vascular proliferation and increase in collateral lumen diameter after femoral artery ligation (FAL). (**a**) The line graph depicts the relative perfusion in hindlimbs of wildtype, iso-antibody-treated (isotype), and γδ T cell-depleted (γδT cell-depl.) mice at baseline, immediately after femoral artery ligation (aFAL), and at days 3 and 7 after the induction of arteriogenesis. *n* = 5 per group. Data are means ± SEM; day 3: °: γδT cell-depl. vs. isotype (*p* < 0.001) and γδT cell-depl. vs. wildtype (*p* < 0.01); day 7: #: γδT cell-depl. vs. isotype (*p* < 0.0001) and γδT cell-depl. vs. wildtype (*p* < 0.0001); two-way ANOVA with Bonferroni’s multiple comparison test. (**b**) Representative laser-Doppler images of hindlimb perfusion directly after FAL and at day 7 (γδT cell-depl. vs. isotype). The flux scale visualizes flow speeds (red = high flow; blue = low flow). Scatter plots display (**c**) the relative number of proliferating vascular cells of all vascular cells and (**d**) the inner luminal diameter of collaterals in adductor muscle (in µm). Wildtype: *n* = 3, isotype: *n* = 4 and γδT cell-depl.: *n* = 5. Two collaterals on three sections per muscle were analyzed. The dotted line in panels (**c**,**d**) indicates the average number of proliferating cells of collaterals, respectively the inner luminal diameters in sham-operated muscles. Data are means ± SEM; * *p* < 0.05, ns = non-significant, two-way ANOVA with Bonferroni’s multiple comparison test. (**e**) Representative immunofluorescence images of collateral arteries (merged, (**left**); single stains, (**right**)) from wildtype (**upper**) and γδT cell-depleted (**lower**) mice. DAPI (blue) marks nuclei, ACTA2 (green) smooth muscle cells, BrdU (red) proliferating cells, and CD31 (white) endothelial cells. Scale bar 20 µm.

On day 7 after FAL, γδ T cell-depleted mice showed significantly lower perfusion recovery than both untreated wildtype and isotype groups in laser-Doppler imaging (Figure 1a,b). Depletion was confirmed by a significant reduction in γδ T cells compared to controls, while no substantial changes were observed in other major leukocyte populations. (see Supplementary Figure S5a–c and Ref. [34]).

### 3.3. γδ T Cell Depletion Impairs Vascular Cell Proliferation and Mitigates Collateral Diameter Growth

To quantify proliferation, we counted BrdU-positive vascular cells (BrdU^+^/CD31^+^ and BrdU^+^/ACTA2^+^), representing proliferating endothelial and smooth muscle cells, and assessed their percentage of all CD31-positive or ACTA2-positive vascular cells, respectively.

BrdU immunofluorescence staining of tissue samples harvested on day 7 after FAL revealed significantly fewer proliferating vascular cells in γδ T cell-depleted mice compared to both control groups, accompanied by significantly smaller collateral lumen diameters (Figure 1c–e).

### 3.4. γδ T Cell Depletion Interferes with Perivascular M2-like-Polarized Macrophage Accumulation

As perivascular macrophage accumulation is pivotal for vascular remodeling and endothelial cell proliferation, we quantified the number of perivascular macrophages and their polarization state.

**Figure 2:**
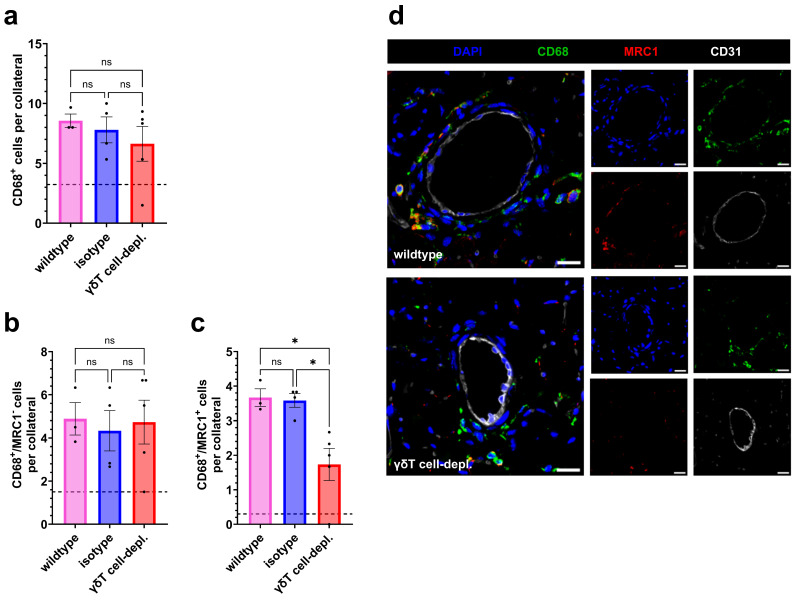
γδ T cell depletion reduces M2-like perivascular macrophage presence but does not alter total and M1-like macrophage accumulation on day 7 after femoral artery ligation (FAL). The scatter plots depict (**a**) the number of macrophages (CD68^+^), (**b**) the number of M1-like polarized (CD68^+^/MRC1^−^) macrophages, and (**c**) the number of M2-like polarized (CD68^+^/MRC1^+^) macrophages in the perivascular space of collaterals in wildtype, isotype, and γδ T cell-depleted (γδT cell-depl.) mice 7 days after FAL. Wildtype: *n* = 3, isotype: *n* = 4 and γδT cell-depl.: *n* = 5. Two collaterals on three sections per muscle were analyzed. The dotted line indicates the average number of cells surrounding collaterals in sham-operated muscles. Data are means ± SEM; * *p* < 0.05, ns = non-significant, two-way ANOVA with Bonferroni’s multiple comparison test. (**d**) Representative immunofluorescence images of collateral arteries (merged, (**left**); single stains, (**right**)) from wildtype (**upper**) and γδT cell-depleted (**lower**) mice show CD68 and MRC1 labeled macrophages (anti-CD68, green; anti-MRC1, red), endothelial cells (anti-CD31, white), and nuclei (DAPI, blue). Scale bars: 20 µm.

At day 7, no differences regarding the number of perivascular macrophages present were noted between all experimental groups (Figure 2a,d). However, when investigating the subpopulations, we found that the number of M2-like macrophages (MRC1^+^) was significantly reduced in γδ T cell-depleted mice compared to both control groups, while the number of M1-like macrophages was not altered (Figure 2b–d).

### 3.5. Early Platelet–Leukocyte Aggregate Formation Is Preserved After γδ T Cell Depletion

Platelet–neutrophil aggregate (PNA) formation represents an early event in arteriogenesis, a prerequisite for mast cell degranulation, along with increased subsequent leukocyte infiltration into the collateral perivascular space [3]. Monocytes activated by platelet aggregation (MPA formation) travel to the endothelium and differentiate in the perivascular space into macrophages [35,36]. Flow cytometric analysis was performed to evaluate the impact of γδ T cell depletion on PNA and MPA formation and the presence of neutrophil and monocyte populations in peripheral blood.

**Figure 3:**
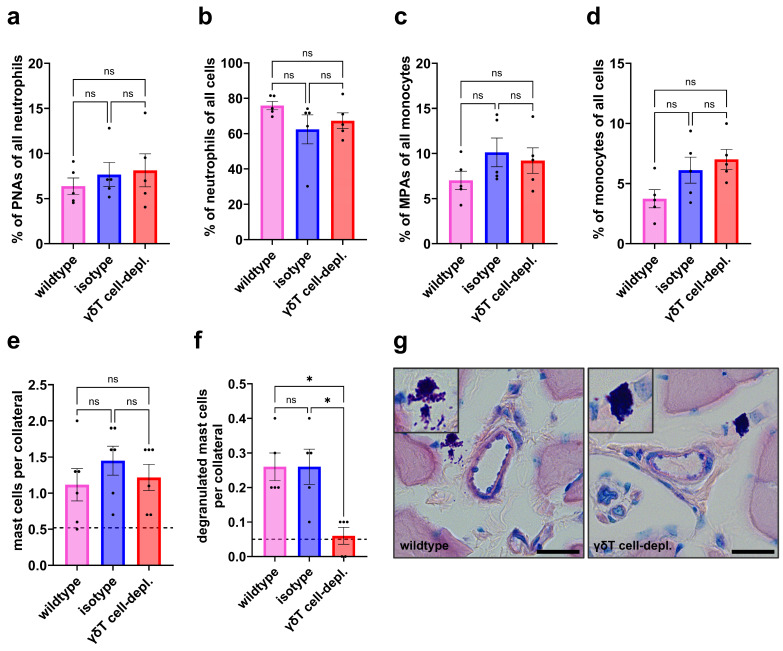
γδ T cell depletion significantly reduces mast cell degranulation on day 1 after femoral artery ligation (FAL), while platelet aggregate formation and mast cell recruitment remain unaffected. The scatter plots (**a**–**d**) show flow cytometry analysis of whole blood on day 1 after FAL: (**a**) platelet-neutrophil aggregates (PNAs, % of neutrophils), (**b**) neutrophils (% of all cells), (**c**) monocyte-platelet aggregates (MPAs, % of monocytes), (**d**) monocytes (% of all cells). The scatter plots (**e**) show the number of perivascular mast cells and (**f**) the number of degranulated perivascular mast cells in wildtype, isotype, and γδ T cell-depleted (γδT cell-depl.) mice on day 1 after FAL. For panels: (**a**–**f**), *n* = 5 in all groups. For panels (**e**,**f**), two collaterals on five sections per muscle were analyzed. The dotted line indicates the average number of cells surrounding collaterals in sham-operated muscles. Data for panels (**a**–**f**) are means ± SEM; * *p* < 0.05, ns = non-significant, statistical analysis was performed using one-way ANOVA with Bonferroni’s multiple comparison test. (**g**) Representative images of Giemsa-stained perivascular mast cells: in wildtype mice (**left**) showing a degranulated mast cell and in γδT cell-depl. mice (**right**) showing a dormant mast cell around a growing collateral artery on day 1 after FAL, inserts show magnified images of representative mast cells. Scale bars: 20 µm.

On day 1 after induction of arteriogenesis by FAL, flow cytometric analysis of whole blood revealed no differences among groups in PNA or MPA formation (Figure 3a,c). The proportions of blood neutrophils and monocytes relative to total leukocytes also did not differ between γδ T cell-depleted and control group mice (Figure 3b,d).

### 3.6. γδ T Cell Depletion Diminishes Perivascular Mast Cell Degranulation Without Affecting Mast Cell Recruitment

Mast cell recruitment and subsequent degranulation constitute the next steps in the arteriogenic cascade and are essential for the inflammatory response to increased shear stress. To compare these processes between groups, Giemsa staining was performed on day 1 after induction of arteriogenesis by FAL.

The staining revealed a comparable number of perivascular mast cells across all groups, while significantly fewer degranulated mast cells were detected in γδ T cell-depleted mice compared to the control groups (Figure 3e–g).

### 3.7. Neutrophil Extravasation into the Perivascular Space Is Unaffected by γδ T Cell Depletion

In the process of arteriogenesis, mast cell activation is dependent on reactive oxygen species (ROS), which are produced by platelet-activated neutrophils extravasating into the perivascular space during early inflammation [7]. Since our data indicated reduced mast cell degranulation in the γδ T cell-depleted group, we examined whether this effect was due to impaired neutrophil extravasation. To assess neutrophil infiltration, myeloperoxidase (MPO) staining was performed.

**Figure 4:**
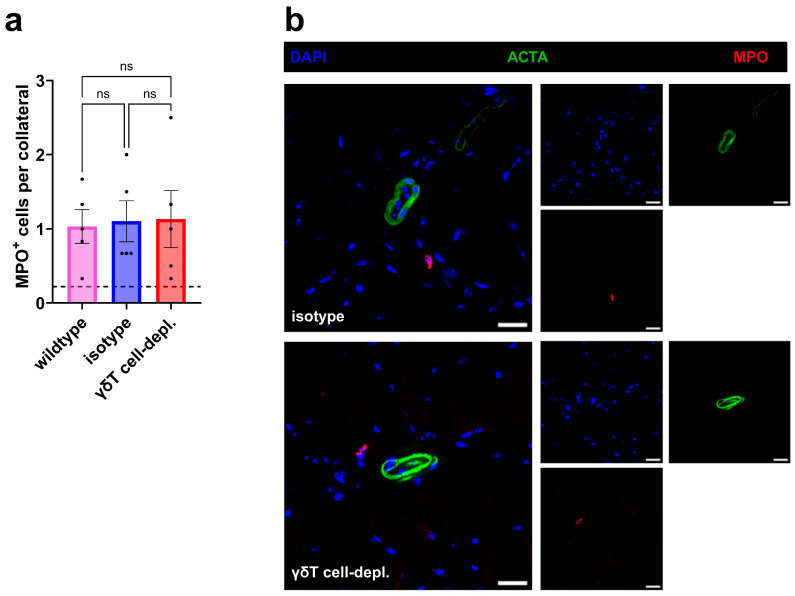
γδ T cell depletion does not alter perivascular neutrophil accumulation. The scatter plot (**a**) displays the numbers of neutrophils (MPO^+^ cells) in the perivascular space of collateral arteries of wildtype, isotype, and γδ T cell-depleted (γδT cell-depl.) mice on 1 day after femoral artery ligation (FAL). *n* = 5 in all groups. Two collaterals on three sections per muscle were analyzed. The dotted line indicates the average number of cells surrounding collaterals in sham-operated muscles. Data are means ± SEM, ns = non-significant, one-way ANOVA with Bonferroni’s multiple comparison test was used for statistical analysis. (**b**) Representative immunofluorescence images of growing collateral arteries on day 1 after FAL (merged, (**left**); single stains, (**right**)) from isotype (**upper**) and γδT cell-depl. (**lower**) mice show perivascular neutrophils (anti-MPO, red), smooth muscle cells (anti-ACTA2, green), and nuclei (DAPI, blue). Scale bars: 20 µm.

Immunofluorescence staining on day 1 after FAL for MPO^+^ cells demonstrated similar perivascular neutrophil numbers in γδ T cell-depleted, isotype, and wildtype mice (Figure 4a,b).

### 3.8. IFNγ Is Essential for Early Mast Cell Degranulation but Interferes with Late Perivascular M2-like Macrophage Accumulation

**Figure 5:**
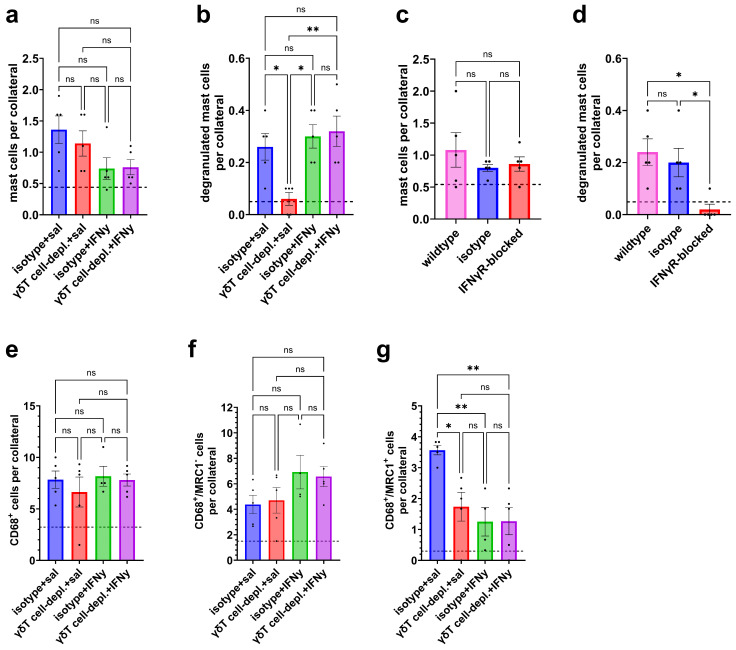
IFNγ administration rescues early mast cell degranulation but impairs late M2-like macrophage polarization. The scatter plots show (**a**) the number of all perivascular mast cells and (**b**) degranulated perivascular mast cells on day 1 after femoral artery ligation (FAL) in isotype and γδ T cell-depleted (γδT cell-depl.) mice either treated with IFNγ or saline (sal). The scatter plots (**c**) show the number of all perivascular mast cells and (**d**) degranulated perivascular mast cells in wildtype, isotype, and IFNγ receptor-blocked (IFNγR blocked) mice on day 1 after FAL. For panels (**a**–**d**): *n* = 5 per group. Two collaterals on five sections per muscle were analyzed. The dotted line indicates the average number of cells surrounding collaterals in sham-operated muscles. Data are presented as means ± SEM. * *p* < 0.05, ** *p* < 0.01, ns = non-significant; two-way ANOVA with Bonferroni’s multiple-comparison test was used for statistical analysis. The scatter plots show (**e**) the number of macrophages (CD68^+^), (**f**) the number of M1-like polarized (CD68^+^/MRC1^−^) macrophages and (**g**) the number of M2-like polarized (CD68^+^/MRC1^+^) macrophages on day 7 after FAL in isotype and γδ T cell-depleted (γδT cell-depl.) mice either treated with IFNγ or saline (sal). For panels (**e**–**g**): *n* = 5 per group, except isotype + IFNγ: *n* = 4. Two collaterals on three sections per muscle were analyzed. The dotted line indicates the average number of cells surrounding collaterals in sham-operated muscles. Data are presented as means ± SEM. * *p* < 0.05, ** *p* < 0.01, ns = non-significant; two-way ANOVA with Bonferroni’s multiple-comparison test was used for statistical analysis.

γδ T cells produce various cytokines, among which IFNγ is a key mediator frequently implicated in inflammation-dependent mechanisms [37,38]. Based on increased Ifng (encoding IFNγ) mRNA expression in γδ T cells of wildtype mice observed following induction of arteriogenesis by femoral artery ligation (see Supplementary Figure S6 and Ref. [34]), γδ T cell-depleted mice were treated with exogenous IFNγ to investigate whether γδ T cell-derived IFNγ is essential for mediating pro-arteriogenic effects. Our results demonstrated that IFNγ administration rescued the mast cell degranulation deficit observed in γδ T cell-depleted mice at day 1 to levels of isotype mice without significantly affecting perivascular mast cell accumulation, although a tendency towards a reduced number of mast cells was observed (Figure 5a,b).

To assess the general relevance of IFNγ in mast cell degranulation during the early phases of arteriogenesis, we treated mice with an IFNγ receptor blocker. Systemic IFNγ receptor blockage did not alter mast cell recruitment but completely inhibited mast cell degranulation in wildtype mice, accounting for the relevance of IFNγ in mast cell activation during the process of arteriogenesis (Figure 5c,d).

Furthermore, we investigated the impact of IFNγ administration on macrophage polarization in iso-antibody-treated and γδ T cell-depleted mice. Perivascular macrophage phenotypes were quantified on day 7 via immunofluorescence staining. Administration of IFNγ showed no effect on the total numbers of CD68^+^ macrophages. Moreover, the number of M1-like macrophages was also unimpaired, although there was a tendency to an increased number of perivascular M1-like macrophages in IFNγ-treated isotype and γδ T cell-depleted mice. However, daily administration of IFNγ significantly reduced the number of M2-like macrophages in the perivascular space in γδ T cell-depleted as well as isotype mice compared to saline-treated controls (Figure 5e–g).

### 3.9. γδ T Cell Subsets Dynamically Reprogram During Arteriogenesis

Different γδ T-cell subpopulations can produce distinct, and in some cases opposing, cytokines to regulate immunological processes [18,39,40,41]. To assess the involvement of these subsets during arteriogenesis, we performed immunohistochemical staining of mice adductor muscle samples, as well as flow cytometric analysis of whole blood on days 1 and 3 after induction of arteriogenesis by FAL.

**Figure 6:**
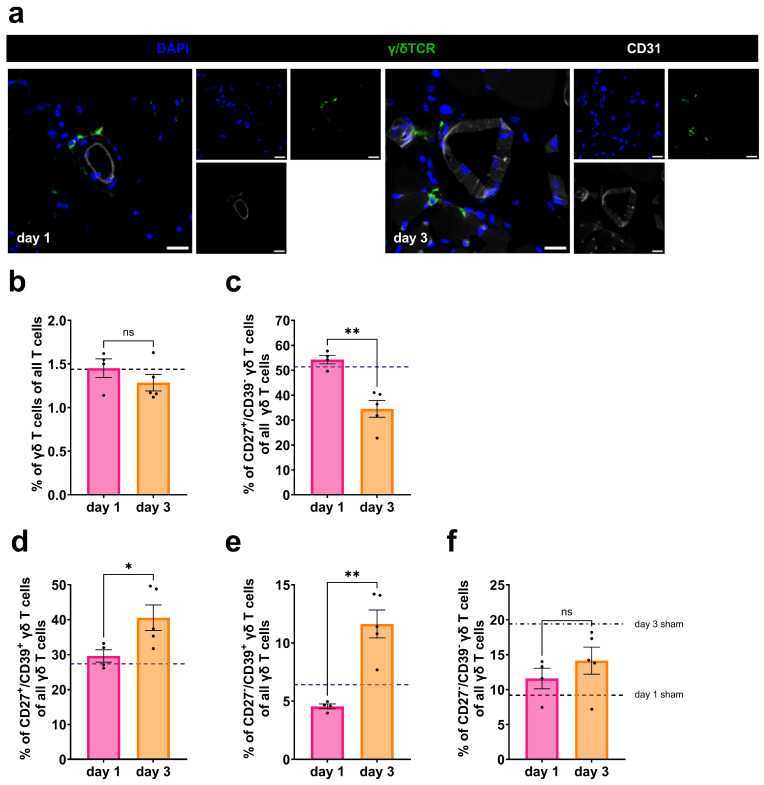
γδ T cell subsets reprogram during arteriogenesis. (**a**) Representative immunofluorescence images of collateral arteries surrounded by γδ T cells (merged, (**left**); single stains, (**right**)) from wildtype mice on day 1 (**left panel**) and day 3 (**right panel**) after femoral artery ligation (FAL). DAPI (blue) stains nuclei, γ/δTCR (green) stains γδ T cells, and CD31 (white) stains endothelial cells. Scale bars: 20 µm. The scatter plots show the percentage of (**b**) γδ T cells of all T cells, (**c**) CD27^+^/CD39^−^ (IFNγ-associated) γδ T cells, (**d**) double-positive (CD27^+^/CD39^+^) regulatory γδ T cells, (**e**) CD27^−^/CD39^+^ (IL-10-associated) γδ T cells, and (**f**) double-negative (CD27^−^/CD39^−^) non-active γδ T cells in peripheral blood of wildtype mice on day 1 and day 3 after FAL. For panels (**b**–**f**): day 1 after FAL: *n* = 4, day 3 after FAL: *n* = 5. The dotted line represents average cell percentages in sham-operated wildtype mice. Data are means ± SEM; * *p* < 0.05, ** *p* < 0.01, ns = non-significant; statistical analysis was performed by Student’s *t*-test.

Immunofluorescence staining showed the presence of γδ T cells in the perivascular space at different time points after induction of arteriogenesis (Figure 6a). γδ T-cell subtypes were defined by CD27 and CD39 expression to discriminate between IFNγ-producing (CD27^+^/CD39^−^), regulatory (CD27^+^/CD39^+^), anti-inflammatory IL-10-associated (CD27^−^/CD39^+^), and non-active (CD27^−^/CD39^−^) populations. Here, the term ‘regulatory’ refers to immunomodulatory γδ T cell subsets characterized by CD27 and CD39 co-expression, which have been associated with immunomodulatory function, and should be distinguished from classical Foxp3^+^ regulatory T cells of the αβ lineage [42,43]. Flow cytometric analysis revealed dynamic temporal changes in γδ T-cell subtype composition following FAL. While the overall proportion of γδ T cells remained constant, CD27^+^/CD39^−^ γδ T cells predominated on day 1, whereas by day 3, CD39^+^ subsets increased (Figure 6b–f).

### 3.10. IL-10 Substitution Favors M2-like Polarization and Enhances Perfusion Recovery

IL-10 is a predominantly anti-inflammatory cytokine with key roles in immune regulation. In γδ T cells, the expression of the cluster of differentiation 39 (CD39) coincides with high expression levels of IL-10 [42]. We hypothesized that IL-10 from anti-inflammatory acting γδ T cells was the missing immunosuppressing cytokine needed to resolve inflammation and drive collateral growth in the late phase of arteriogenesis. Based on our flow cytometry data indicating a shift toward IL-10-producing γδ T cells, mice were treated with IL-10, administered intravenously on day 3 after FAL.

**Figure 7:**
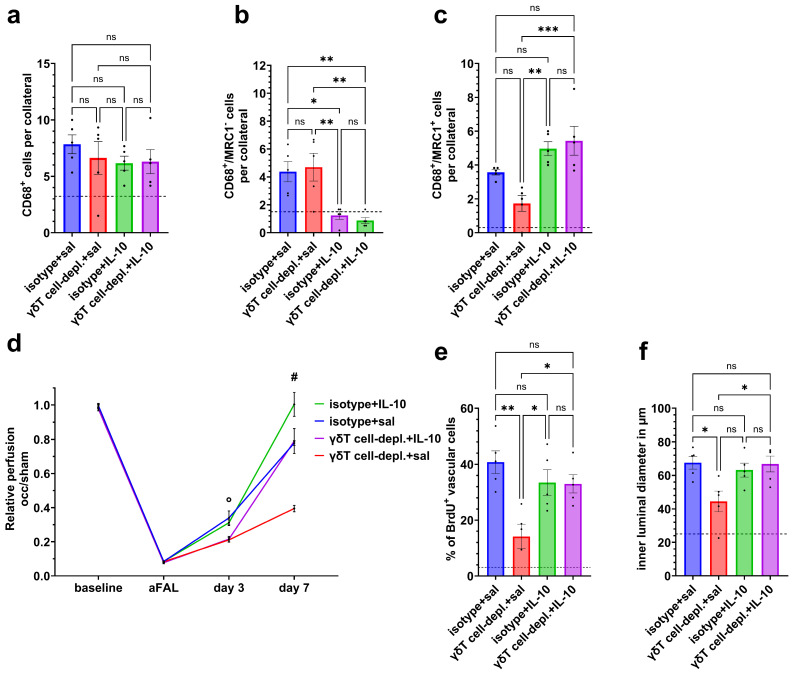
IL-10 administration rescues macrophage polarization along with vascular cell proliferation and perfusion recovery in γδ T cell-depleted mice. The scatter plots visualize the numbers of (**a**) macrophages (CD68^+^), (**b**) M1-like (CD68^+^/MRC1^−^) macrophages and (**c**) M2-like (CD68^+^/MRC1^+^) macrophages in isotype and γδT cell-depleted (γδT cell-depl.) mice treated with IL-10 or saline (sal) on day 7 after femoral artery ligation (FAL). The dotted line represents the average number of cells in sham muscles. The line graph (**d**) shows the relative hindlimb perfusion in isotype and γδT cell-depl. mice treated with IL-10 or saline (sal) at baseline, immediately after FAL, on day 3, and on day 7. Data are means ± SEM; day 3: °: γδT cell-depl. + sal vs. isotype + sal (*p* < 0.05), γδT cell-depl. + IL-10 vs. isotype + sal (*p* < 0.05), day 7: #: isotype + IL-10 vs. isotype + sal (*p* < 0.0001), γδT cell-depl. + sal vs. isotyp + sal (*p* < 0.0001), γδT cell-depl. + sal vs. isotype + IL-10 (*p* < 0.0001), γδT cell-depl. + IL-10 vs. isotype + IL-10 (*p* < 0.0001), and γδT cell-depl. + IL-10 vs. γδT cell-depl. + sal (*p* < 0.0001). The scatter plots show (**e**) the relative number of proliferating vascular cells and (**f**) the inner luminal diameter of collaterals in adductor muscle (in µm) of isotype and γδT cell-depl. mice treated with IL-10 or saline (sal) on day 7 after FAL. For panels (**a**–**f**), *n* = 5 per group. For panels (**a**–**c**), two collaterals on three sections per muscle were analyzed. The dotted line indicates the average number of cells surrounding collaterals in sham-operated muscles. For panels (**e**,**f**), two collaterals on three sections per muscle were analyzed. The dotted line indicates the average number of proliferating cells in collaterals, respectively the inner luminal diameter in sham-operated muscles. Data (**a**–**c**) and (**e**,**f**) are means ± SEM; * *p* < 0.05, ** *p* < 0.01, *** *p* < 0.001, ns = non-significant; two-way ANOVA with Bonferroni’s multiple comparison test was used for statistical analysis.

While total macrophage numbers remained unchanged, IL-10 promoted the polarization toward M2-like macrophages and reduced M1-like populations in the perivascular regions of growing collaterals (Figure 7a–c).

IL-10 treatment, moreover, improved the perfusion recovery by day 7 after FAL in both γδ T cell-depleted and isotype control mice compared with saline-treated controls (Figure 7d).

Exogenous IL-10 administration also rescued the proliferation of vascular cells, as shown by immunohistochemical BrdU staining, as well as luminal diameter sizes in γδ T cell-depleted mice (Figure 7e,f).

## 4. Discussion

In this study, we define a previously unrecognized, stage-specific role for γδ T cells in arteriogenesis in a murine hind-limb model. Whereas collateral growth and perfusion recovery were preserved in αβ T cell-deficient mice, depletion of γδ T cells markedly impaired arteriogenesis, as evidenced by reduced perfusion recovery, diminished vascular cell proliferation, and smaller collateral diameters. Mechanistically, we identify distinct γδ T cell subsets that gain prominence sequentially during collateral remodeling. Early arteriogenesis was characterized by the emergence of a CD27^+^/CD39^−^ IFNγ-producing γδ T cell population, and proof-of-principle rescue experiments demonstrated that IFNγ, potentially γδ T cell-derived, is essential for mast cell degranulation. At later stages, CD27^−^/CD39^+^ IL-10-producing and CD27^+^/CD39^+^ regulatory γδ T cells became predominant. In γδ T cell-depleted mice, IL-10 administration supported M2-like (MRC1^+^) macrophage polarization and a sustained vascular cell proliferation, suggesting a relevant role for γδ T cells in supplying or supporting production of IL-10. Together, these findings establish γδ T cells as critical regulators of collateral artery growth and propose a temporally coordinated involvement of multiple γδ T cell subsets during arteriogenesis.

Previous studies have reported conflicting roles for T cells in arteriogenesis, with some suggesting a contribution to collateral artery remodeling and others finding T cells to be dispensable [11,44,45,46]. In this context, we first evaluated the contribution of conventional αβ T cells and found that their absence did not influence collateral growth or perfusion recovery in this hindlimb model of arterial ligation. Notably, this lack of a detectable contribution of αβ T cells in our model may be specific to the experimental conditions employed and does not exclude a potential role for these cells in other contexts of collateral vessel growth. In contrast, depletion of γδ T cells profoundly impaired arteriogenesis, as evidenced by reduced perfusion recovery, diminished vascular cell proliferation, and smaller collateral artery diameters. Taken together with our findings, the discrepancies in the literature are best explained by subset-specific effects, suggesting that γδ T cells, but not αβ T cells, are critical regulators of collateral artery growth. The divergent results may also be attributable, at least in part, to differences in experimental approaches and species- or strain-dependent effects.

M2 macrophages are a key determinant of the extent of arteriogenesis and a central driver of vascular cell proliferation and collateral maturation during the inflammation-resolving phase [47,48,49,50,51]. Macrophages supply effector molecules essential for vascular cell proliferation and undergo a characteristic shift from a pro-inflammatory, M1-like phenotype to a regenerative, M2-like phenotype, thereby contributing to the resolution of inflammation and subsequent arterial remodeling during progressed stages of arteriogenesis [49]. In γδ T cell-depleted mice, overall perivascular macrophage recruitment remained unchanged on day 7 after FAL; however, we observed a significant reduction in M2-like polarized macrophages (MRC1^+^) compared to the control groups. This suggests that γδ T cells influence macrophage polarization by promoting the inflammatory-to-proliferative transition of macrophages during the process of collateral artery growth rather than affecting their perivascular accumulation.

To determine whether γδ T cell depletion affects early arteriogenic processes, we investigated mast cell recruitment, activation, and influential upstream events. Mast cell activation is indispensable during the early phase of arteriogenesis, occurring within 24–48 h after vascular occlusion [6]. In our model, γδ T cell depletion led to a marked reduction in mast cell degranulation, while mast cell recruitment itself remained unchanged. These findings indicate that γδ T cells regulate mast cell activation either directly or indirectly.

Previous reports have identified oxygen radicals generated by neutrophils, which are activated by platelet-neutrophil aggregate (PNAs) formation, as the primary trigger for mast cell degranulation during early arteriogenesis [6]. In addition, γδ T cells are known to modulate neutrophil activity in immune-dependent responses [21,52]. Importantly, however, γδ T cell depletion in our arteriogenesis model did not affect inflammatory events necessary for mast cell activation, as PNA formation as well as neutrophil infiltration into the perivascular space were preserved. These findings indicate that γδ T cells primarily regulate mast cell activation directly rather than through the formation of PNAs or the initial recruitment of neutrophils.

Based on the preserved upstream inflammatory recruitment but impaired mast cell degranulation observed in γδ T cell-depleted mice, we sought to define the effector mechanism linking γδ T cells to mast cell activation. Previous studies have reported that γδ T cell-derived cytokines modulate mast cell function, with IFNγ emerging as a predominant effector cytokine that may prime mast cells either through direct receptor engagement or indirectly by regulating neutrophil activity required for mast cell degranulation [37,53,54,55,56,57,58,59]. PCR analysis showed heightened expression of IFNγ mRNA in operated mice compared to sham-operated controls. Therefore, γδ T cell-derived IFNγ may act synergistically with neutrophil-derived reactive oxygen species (ROS) or influence neutrophil priming for ROS production [53,60,61,62,63]. We hypothesized that the absence of γδ T cell-derived IFNγ during early arteriogenesis impairs mast cell responsiveness. To test this hypothesis, we performed rescue experiments in γδ T cell-depleted mice using IFNγ. Impaired mast cell activation in these animals was restored by exogenous IFNγ administration and completely abrogated by IFNγ receptor blockade. Together, these results identify IFNγ as a key facilitator of mast cell responsiveness during early collateral artery growth. The observation that IFNγ administration attenuated the effects of prior γδ T cell depletion suggests that γδ T cells may contribute to IFNγ-mediated signaling or to upstream regulatory mechanisms within this pathway. However, these data do not provide definitive evidence that γδ T cells represent the physiological or predominant source of IFNγ in this context.

We further explored whether IFNγ signaling could also account for the later effects of γδ T cell depletion, particularly with respect to macrophage polarization. IFNγ is well established as a potent regulator of macrophage phenotype, predominantly promoting polarization toward an M1-like state [64,65]. However, in our experimental setup, IFNγ supplementation reduced M2-like (MRC1^+^) macrophage polarization further in γδ T cell-depleted mice and reduced M2-like (MRC1^+^) macrophages in the isotype control group, underscoring its pro-inflammatory bias. This suggests that additional γδ T cell-derived signals are required for complete perfusion recovery and hints at the involvement of a different, more anti-inflammatory acting γδ T cell subset [20,42,66].

The diverse effects of γδ T cells align with their well-documented functional plasticity, shaped by tissue context and subset composition [18,40]. This plasticity makes them key regulators in diverse immunomodulatory processes and suggests that distinct γδ T cell subtypes may assume different roles at specific stages of arteriogenesis [66,67,68,69]. Both murine and human γδ T cells comprise multiple subpopulations distinguished by clusters of differentiation markers and effector molecule profiles. These differences arise from the expression of distinct γ- and δ-chain isoforms, which vary across species and confer adaptive capacity to respond to microenvironmental cues, for example, by altering their cytokine repertoire [66,67,70,71,72]. One study showed that human Vγ9Vδ2 T cells have potent anti-tumor effects, whereas Vδ1 T cells can dampen anti-tumor immunity and even promote tumor progression [69]. This breadth of functional programs makes γδ T cells versatile mediators of immune-driven processes. Therefore, we hypothesized that the functional diversity of γδ T cells enables distinct subsets to contribute sequentially to different phases of arteriogenesis, initially promoting pro-inflammatory activation and subsequently facilitating resolution and arterial remodeling. Consistent with this hypothesis, we observed temporal reprogramming of γδ T cell subsets during collateral artery growth in flow cytometry analysis. During the early inflammatory phase following femoral artery ligation, CD27^+^CD39^−^ IFNγ-producing γδ T cells predominated, indicating a possible involvement in mast cell activation [43,73]. As the immune response progressed, the γδ T cell compartment shifted toward CD27^−^ CD39^+^ IL-10-producing and CD27^+^CD39^+^ regulatory phenotypes, coinciding with the transition from inflammation to regeneration and the concomitant conversion of macrophages from M1-like to M2-like states [4,42,49,74]. Previous studies have described a context-dependent role for γδ T cells in shaping macrophage polarization, promoting either M1- or M2-like phenotypes depending on the inflammatory setting [19,75,76,77]. Given the essential role of M2 macrophages in arteriogenesis, we therefore hypothesized that γδ T cells drive macrophage polarization toward an M2-like state during collateral growth.

In the literature, IL-10 is established as a central anti-inflammatory cytokine and has been shown to be produced by distinct γδ T cell subsets, limiting inflammation in infectious settings [78,79,80,81]. Beyond γδ T cells, M2-like macrophages represent a well-established source of IL-10 [82,83,84]. Accordingly, we hypothesized that γδ T cells may be involved in the later regenerative phase of arteriogenesis, either through direct IL-10 production or by promoting the shift to M2-like IL-10-producing macrophages. IL-10 not only reduces inflammation but also promotes vascular cell proliferation and orchestrates the resolution phase of immune responses [85]. Moreover, IL-10 can directly promote an anti-inflammatory cascade by driving macrophage polarization toward the M2-like phenotype, which is crucial for effective collateral vessel remodeling and tissue reperfusion [78]. Supplementation with IL-10 in γδ T cell-depleted mice improved hindlimb perfusion and vascular cell proliferation and was associated with an increased proportion of M2-like (MRC1^+^) macrophages during the regenerative phase of arteriogenesis. When considered together with our flow cytometry data, which indicate a shift toward regulatory, IL-10-associated γδ T cell subsets, these findings are consistent with a potential functional link between γδ T cell-mediated regulation and macrophage polarization. However, a direct mechanistic relationship cannot be conclusively established based on the present data and might represent a starting point for further exploration. IL-10 can also act as a vasodilatory agent, explaining the improvement in perfusion recovery in the isotype group, which was not fully representative of the vascular cell growth or expansion of collateral diameters compared to the results of the depleted group [86,87].

The functional importance of IFNγ and IL-10 is supported by the results of our rescue experiments. We show that Ifng (encoding IFNγ) mRNA is increased in γδ T cells following induction of arteriogenesis, and our flow cytometry analysis demonstrates a temporal shift in γδ T cell subtypes expressing CD27 and/or CD39 at different time points. Together, these findings suggest a dual role for γδ T cells in arteriogenesis, with sequential contributions to both the inflammatory initiation and resolution phases. This supports a model in which γδ T cells may contribute to cytokine-mediated regulation, with early subsets associated with IFNγ-related responses promoting mast cell activation and leukocyte recruitment, and later subsets with regulatory features potentially supporting macrophage polarization and collateral remodeling. While γδ T cells may contribute to these processes, additional cellular sources of IFNγ and IL-10 cannot be excluded and may act in part downstream of γδ T cell-mediated signaling. These results help reconcile prior discrepancies in the literature regarding T cell involvement in arteriogenesis and identify γδ T cells as dynamic, context-dependent regulators. Modulation of γδ T cell function may represent a novel strategy to enhance arteriogenesis with clinical relevance and to enable the development of new therapeutic approaches for vascular occlusive disease.

This study has several limitations. Analyses by the primary investigator were not blinded; however, outcome measures were based on predefined quantitative criteria and evaluated by a blinded observer, while animals were allocated to groups in a random manner and carefully matched for sex, age, and body weight to minimize variability. Furthermore, endogenous levels of IFNγ and IL-10 and their cellular sources were not assessed beyond the measurement of increased Ifng mRNA expression in wildtype mice following femoral artery ligation. Therefore, cytokine administration experiments, which were not designed to replicate physiological cytokine concentrations, should be interpreted as proof-of-principle approaches of cytokine-dependent effects rather than definitive evidence of γδ T cell-specific mechanisms. Finally, macrophage phenotypes were defined based on CD68 and MRC1 expression, which does not fully capture the spectrum of macrophage polarization and should be considered an operational approximation. While the murine hindlimb ischemia model provides important mechanistic insights into arteriogenesis, species-specific differences of murine and human γδ T cell subtype compositions may limit the direct translational applicability of these findings [88]. It should also be noted that the approaches used to assess the role of αβ and γδ T cells differ in their underlying mechanisms and biological consequences. In TCRα knockout mice, αβ T cells are absent throughout development, which may lead to compensatory adaptations within the immune system. In contrast, anti-TCRδ-antibody-mediated γδ T cell depletion results in a transient and potentially incomplete reduction of the target population, as newly generated cells can repopulate the compartment. However, in our study, γδ T cell depletion was confirmed at the experimental endpoint, indicating that reduced cell numbers were maintained over the course of the experiment. Moreover, although anti-TCRδ-mediated depletion is an established approach, off-target effects, including transient changes in other lymphocyte populations and Fc receptor-/complement-mediated immune activation, cannot be entirely ruled out [89,90,91]. While our data do not establish definitive causality of the effects of γδ T cell depletion, iso-antibody-treated mice were included throughout to control for the previously described potential side-effects. Despite these limitations, the consistent findings across independent experimental approaches support a regulatory role for γδ T cells in arteriogenesis.

Future work should delineate the roles of distinct γδ T cell subsets and upstream mechanisms guiding their recruitment and activation. Insights from other immune contexts suggest that B cells or related populations may influence γδ T cell polarization during arteriogenesis [92,93,94,95], a topic that should be investigated in further studies. Additionally, characterization of human γδ T cell subsets in patients with coronary or peripheral artery disease may provide insight into temporal changes in subset distribution and their involvement in the perivascular environment. This may help to better understand their role during collateral vessel growth in response to progressive arterial occlusion. From a translational perspective, these findings raise the possibility that temporally coordinated modulation of distinct γδ T cell subsets, potentially promoting sequential inflammatory and regulatory mechanisms, could represent a strategy to stimulate collateral artery growth in patients with relevant arterial stenosis. However, studies using human cell populations and later human test subjects are required to evaluate the feasibility and clinical applicability of such approaches.

## 5. Conclusions

Selective depletion of γδ T cells altered immune responses, reduced vascular cell proliferation, and impaired perfusion recovery, whereas the absence of αβ T cells had no comparable effect in the employed hindlimb model of arterial ligation. Importantly, these effects of γδ T cell depletion could be attenuated or partially reversed by cytokine substitution, underscoring the regulatory capacity of γδ T cells in orchestrating arteriogenesis through direct or indirect supply of key cytokines required for collateral artery growth.

Our data further suggests the presence of functionally distinct γδ T cell subsets that differentially shape the inflammatory milieu during arteriogenesis. CD27^+^/CD39^−^ γδ T cells, characterized by IFNγ production, appear to be critical for early pro-inflammatory activation of immune cells, whereas later-emerging CD39^+^ subsets, capable of producing IL-10 and associated with regulatory function, may contribute to resolution and vascular remodeling. Together, these temporally distinct γδ T cell responses likely cooperate to coordinate immune activation and resolution, thereby promoting collateral artery growth.

## Data Availability

The raw data supporting the conclusions of this article will be made available by the authors on request.

## Supplementary Materials

The following supporting information can be downloaded at: https://www.mdpi.com/article/10.3390/cells15080709/s1, Figure S1. Process of femoral artery ligation; Figure S2. Placement of regions of interest (ROIs) for perfusion analysis; Figure S3. Localization of collateral arteries and preparation of tissue sections; Figure S4. αβ T cell deficiency does not impair perfusion recovery or vascular cell proliferation after FAL; Figure S5. Antibody-mediated depletion markedly reduces γδ T cell frequency, while white blood cell counts stay consistent; Figure S6. Ifng mRNA expression is increased in γδ T cells of wildtype mice following femoral artery ligation.

## Abbreviations

The following abbreviations are used in this manuscript:

αβ T cells	alpha beta T cells
BSA	Bovine serum albumin
BrdU	Bromodeoxyuridine
CD	Cluster of Differentiation
depl	depleted
DNA	Deoxyribonucleic Acid
FAL	Femoral Artery Ligation
γδ T cells	gamma delta T cells
GPIbα	Glycoprotein Ib alpha
IL-10	Interleukin-10
IL-17	Interleukin-17
IFNγ	Interferon gamma
LDI	laser-Doppler imaging
MMF	Midazolam, Medetomidine and Fentanyl
MRC1	Mannose Receptor C-type 1
MPA	Monocyte-Platelet Aggregate
MPO	Myeloperoxidase
NO	Nitric Oxide
ns	not significant
occ	occluded
PBS	Phosphate-Buffered Saline
PCR	Polymerase Chain Reaction
PFA	Paraformaldehyde
PNA	Platelet–Neutrophil Aggregate
RNA	Ribonucleic Acid
ROI	Region Of Interest
SEM	Standard Error of the Mean
TCR	T cell receptor
VWF	Von Willebrand Factor
