# Supplementary material for: Small Subset, Big Impact: Regulatory Function of γδ T Cells in Arteriogenesis
Source: Cells. 2026 Apr 17;15(8):709. doi: 10.3390/cells15080709 (PMC13115087; doi:10.3390/cells15080709)
Supplement: Supplementary file 1 [file cells-15-00709-s001.zip › supplement Small Subset, Big Impact Regulatory Function of gamma delta T cells in Arteriogenesis.pdf]

cells

MDPI

Article

Small Subset, Big Impact: Regulatory Function of γδ T cells in Arteriogenesis

Kira-Sofie Wimmer [1,2], Carolin Baur [1,2], Matthias Kübler [1,2,3,4], Christoph Arnholdt [1,2,5], Konda Kumaraswami [6], Franziska Heim [1,2], Katharina Elbs [1,2], Michael Reha Rohrmoser [1,2], Daphne Merkus [1,7] and Elisabeth Deindl [1,2,*]

1. Institute of Surgical Research at the Walter-Brendel-Centre of Experimental Medicine, University Hospital, Ludwig-Maximilians-Universität München, 81377 Munich, Germany; kira.wimmer@med.uni-muenchen.de (K.-S.W.); carolin.baur@med.uni-muenchen.de (C.B.); christophjohannes.arnholdt@med.uni-heidelberg.de (C.A.); matthias.kuebler@gmail.com (M.K.); franziska.heim@med.uni-muenchen.de (F.H.); katharina.elbs@med.uni-muenchen.de (K.E.); michael.rohrmoser@med.uni-muenchen.de (M.R.R.); daphne.merkus@med.uni-muenchen.de (D.M.); elisabeth.deindl@med.uni-muenchen.de (E.D.)
2. Biomedical Center, Institute of Cardiovascular Physiology and Pathophysiology, Faculty of Medicine, Ludwig-Maximilians-Universität München, 82152 Planegg-Martinsried, Germany
3. Deutsches Zentrum Immuntherapie (DZI) and Comprehensive Cancer Center Erlangen-EMN (CCC ER-EMN), Friedrich-Alexander-Universität Erlangen-Nürnberg (FAU), Erlangen, Germany
4. Department of Oral- and Cranio-Maxillofacial Surgery, Friedrich-Alexander-Universität Erlangen-Nürnberg (FAU), Erlangen, Germany
5. Department of Ophthalmology, University of Heidelberg, 69120 Heidelberg, Germany
6. Immunoregulation section, Laboratory of Molecular Biology and Immunology, National Institute on Aging, 251 Bayview Blvd, Suite 100, Baltimore, MD 21224, USA
7. Division of Experimental Cardiology, Department of Cardiology, Thoraxcenter, Erasmus MC, University Medical Center Rotterdam, Rotterdam, the Netherlands

* Correspondence: elisabeth.deindl@med.uni-muenchen.de (E.D.); Tel.: +49-(0)-89–2180–76504

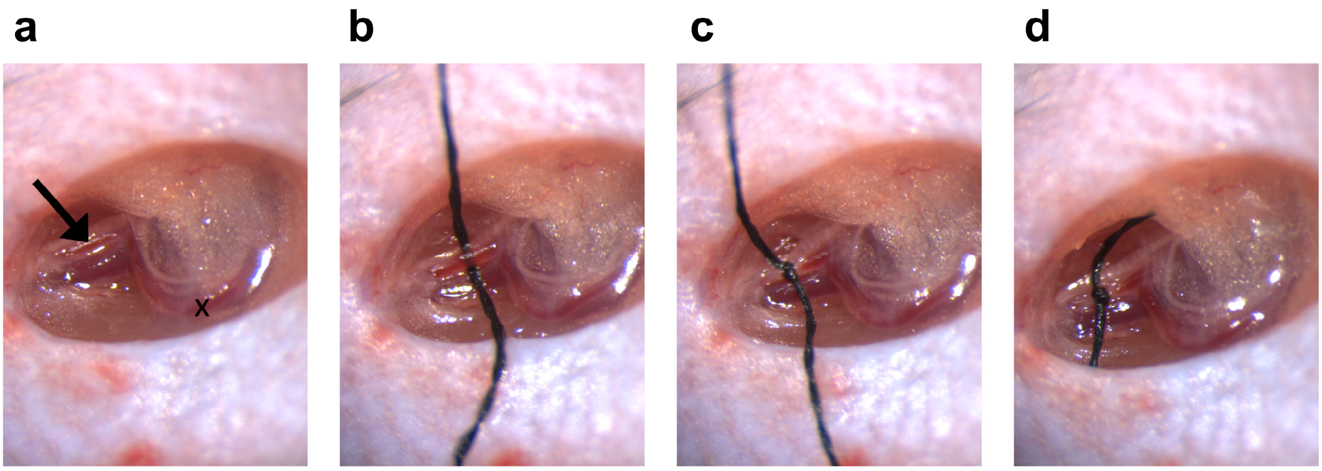

Figure S1. Procedure of femoral artery ligation.

Representative images illustrate the surgical procedure: **(a)** exposure of the femoral artery (arrow) just distal to the profunda femoris artery; **(b)** placement of the ligature thread beneath the artery, carefully sparing the adjacent vein and nerve; **(c)** securing the ligature with a double knot; and **(d)** cutting of the thread before closure of the cutaneous wound by suturing (the sham operation was performed identical but without fastening the thread into a knot).

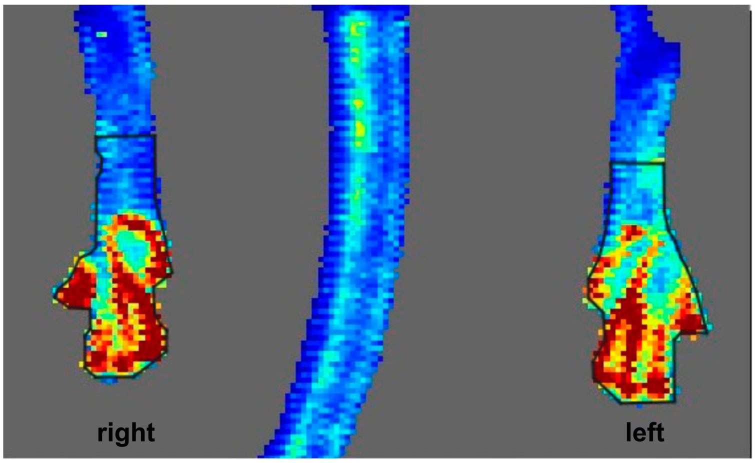

Figure S2. Placement of regions of interest (ROIs) for perfusion analysis.

Representative Laser Doppler imaging (LDI) of mouse hindlimb perfusion prior to femoral artery ligation (FAL). Regions of interest (ROIs) of identical size (0.42cm^2) were placed on the right and left hindlimbs to allow quantitative assessment of perfusion.

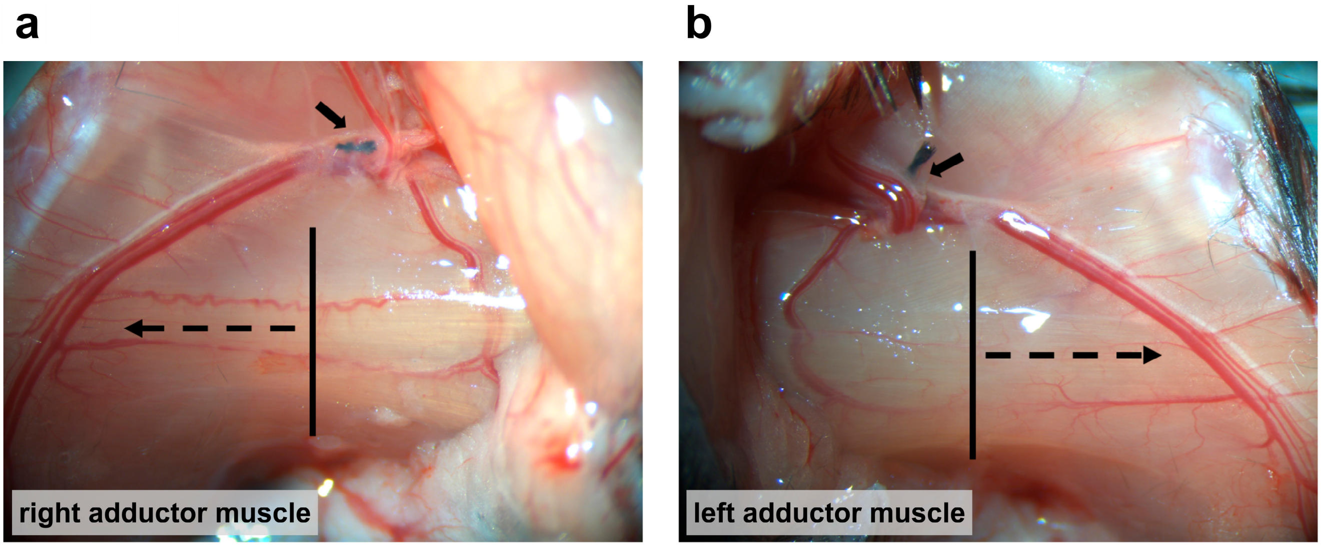

Figure S3. Localization of collateral arteries and preparation of tissue sections.
Representative images of **(a)** the ligated femoral artery in the right adductor muscle and **(b)** the sham-operated, unligated femoral artery in the left adductor muscle. Black arrows indicate the site of femoral artery ligation on the right side and the inlaid surgical thread on the left. The vertical solid line marks the starting point for sectioning, and the dashed horizontal arrow shows the direction in which consecutive tissue slices were collected. On the ligated side **(a)**, enlarged corkscrew-like collaterals are visible, whereas on the sham-operated side **(b)** quiescent, straight, preexisting collaterals are present.

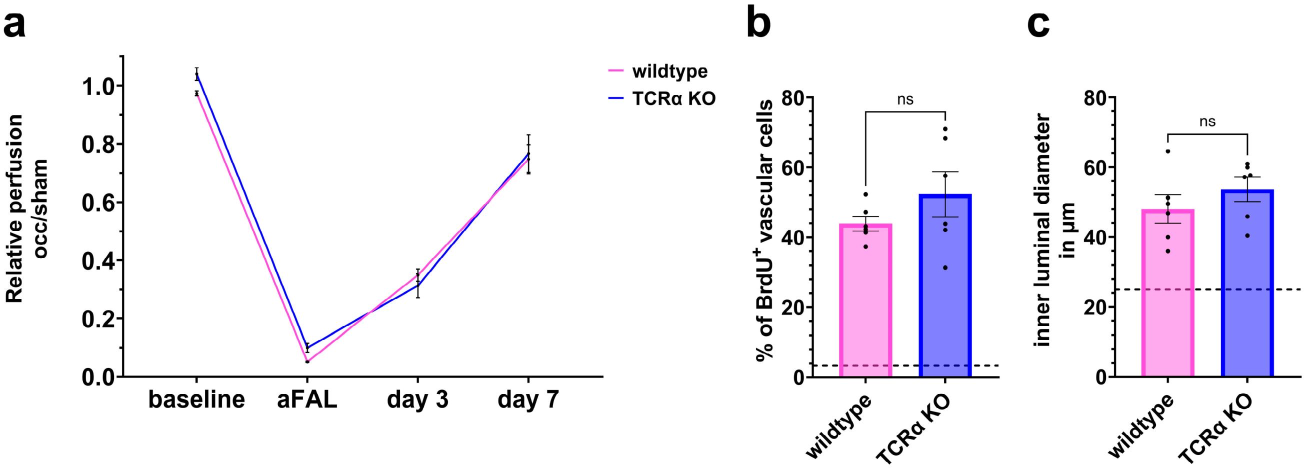

Figure S4. $\alpha\beta$ T cell deficiency does not impair perfusion recovery or vascular cell proliferation after FAL.
(a) Line graph of relative perfusion in hindlimbs of wildtype and TCRα knockout (TCRα KO) mice at baseline, directly after femoral artery ligation (aFAL), and at days 3 and 7; statistical analysis was performed by two-way ANOVA with Bonferroni's multiple comparison test. Bar graphs show **(b)** the absolute number of proliferating vascular cells and **(c)** the inner luminal diameter of growing collaterals in adductor muscles of mice (in µm). The dotted line represents the average in sham-operated tissue samples; *n=6* per group, ns=non-significant, statistical analysis was performed by student's t-test. For graphs a-c: Data are means ± SEM (adapted from Ref [1]).

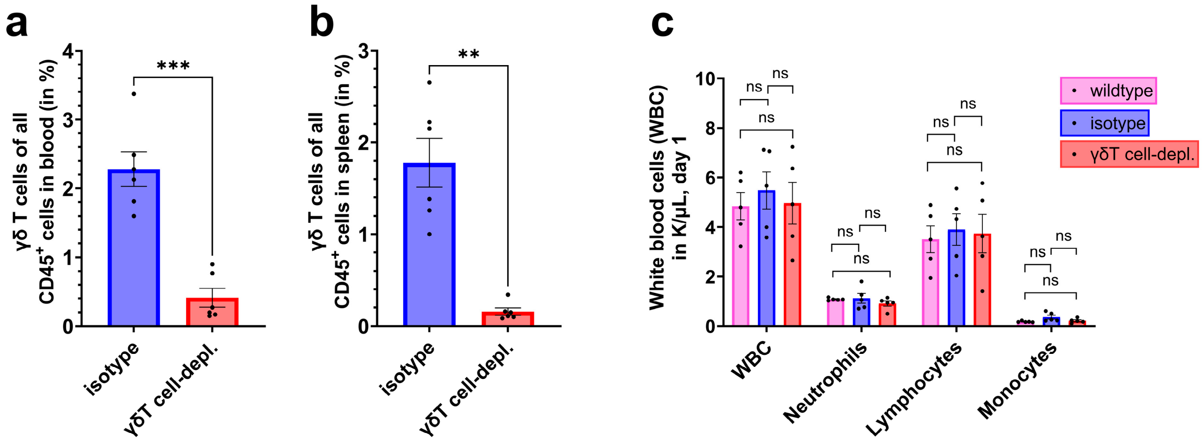

Figure S5. Antibody-mediated depletion markedly reduces γδ T cell frequency, while white blood cell counts stay consistent.

The bar graphs show the percentage of γδ T cells of all CD45⁺ cells in **(a)** blood and **(b)** spleen of C57Bl/J mice on day 7 after femoral artery ligation (FAL) treated with either an anti-TCRδ depleting antibody (γδT cell-depl.) or an iso-antibody (isotype) eight days prior, measured by flow cytometry analysis. Isotype: *n=6*, γδT cell-depl.: *n=6*, **p<0.01, ***p<0.001, statistical analysis was performed by student's t-test. Data are means ± SEM (adapted from Ref. [1]). The bar graph **(c)** depicts the number of all white blood cells (WBC), neutrophils, lymphocytes, and monocytes in K/µL in whole blood collected 24 h after femoral artery ligation in γδ T cell-depleted (γδT cell-depl.) vs. isotype and wildtype control mice, analyzed by differential blood count. *n=5* per group, ns=non-significant. Statistical analysis was performed by two-way ANOVA with Bonferroni's multiple comparison test. Data are means ± SEM.

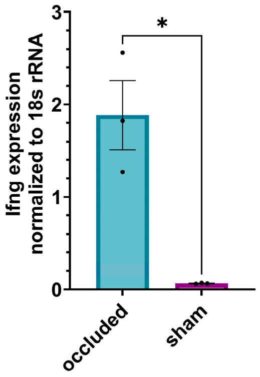

Figure S6. Ifng mRNA expression is increased in γδ T cells of wildtype mice following femoral artery ligation.

The bar graph shows the results of quantitative real-time PCR of Ifng (encoding IFNγ) mRNA expression in γδ T cells normalized to 18S rRNA of wildtype mice after femoral artery ligation (occluded) vs. after sham operation (sham = operation performed without ligating the femoral artery). Per group: *n=3*, *p<0,05, statistical analysis was performed by student's t-test. Data are means ± SEM (adapted from Ref. [1]).

References

1. Kumaraswami, K., *Relevance of Lymphocytes in Collateral Artery Growth (Arteriogenesis)*. 2022, LMU: Walter Brendel Centre of Experimental Medicine (WBex).
